# Aerobic exercise reduces lactate accumulation and improves cardiac function after myocardial infarction

**DOI:** 10.3389/fmed.2026.1816790

**Published:** 2026-04-20

**Authors:** Ze Chen, Jimin Du, Shengkai Zuo, Chunxiao Wan

**Affiliations:** 1 Department of Physical and Rehabilitation Medicine, Tianjin Medical University General Hospital, Tianjin, China; 2 College of Rehabilitation Medicine, Tianjin Medical University, Tianjin, China; 3 School of Pharmacy, Tianjin Medical University, Tianjin, China

**Keywords:** aerobic exercise, cardiac dysfunction, fibroblasts, lactate, myocardial infarction

## Abstract

**Objective:**

This study aimed to investigate the role of lactate in the progression of cardiac dysfunction after myocardial infarction (MI) and clarify the effect of aerobic exercise (AE) on improving post-infarction cardiac function by regulating lactate metabolism, so as to provide experimental evidence for the clinical improvement of cardiac function after MI.

**Methods:**

NIH 3 T3 mouse embryonic fibroblasts and C57BL/6 J mice were used as research subjects. *In vitro*, fibroblasts were treated with different concentrations of lactate, and the activation state of fibroblasts was evaluated by detecting the expression levels of Collagen I (COL I) and *α*-Smooth Muscle Actin (α-SMA). *In vivo*, four mouse models (SED-SHAM, AE-SHAM, SED-MI, and AE-MI) were established. Cardiac function was assessed by echocardiography for left ventricular ejection fraction (LVEF) and left ventricular fractional shortening (LVFS). Lactate levels in cardiac tissue and serum were detected using a lactate assay kit, and serum metabolite changes mediated by lactate were analyzed via metabolomics technology.

**Results:**

*In vitro* experiments confirmed that lactate could significantly induce fibroblast activation. Metabolomics results showed that elevated lactate levels after MI led to abnormal accumulation of arachidonoyl carnitine. *In vivo* experiments revealed that lactate levels in cardiac tissue and serum were significantly increased, while LVEF and LVFS were decreased in the SED-MI group. In contrast, lactate levels in cardiac tissue and serum were reduced, and LVEF and LVFS were elevated in the AE-SHAM group. Lactate levels in cardiac tissue and serum of the AE-MI group were lower than those of the SED-MI group but higher than those of the AE-SHAM group, and the LVEF and LVFS of the AE-MI group were significantly higher than those of the SED-MI group.

**Conclusion:**

This study confirmed that lactate accumulation is involved in the pathological process of cardiac dysfunction after myocardial infarction, and AE can improve cardiac function after MI by reducing lactate levels in cardiac tissue and serum. These findings provide new experimental evidence for the prevention and treatment of post-infarction cardiac dysfunction by targeting lactate metabolism, and also offer theoretical support for the application of AE in cardiac rehabilitation.

## Introduction

Myocardial infarction (MI) has a high morbidity and mortality rate worldwide, imposing a severe financial burden on the global society. It is reported that approximately 7 million people suffer from MI each year with a mortality rate of up to 33.3%, posing a major threat to public health (1). After MI, the heart undergoes a series of complex pathophysiological changes, among which myocardial fibrosis is one of the key factors affecting the recovery of cardiac function (2).

Subsequent to MI, the heart initiates a series of complex and orderly remodeling processes, and cardiac fibroblasts exert distinct roles at different stages after MI (2). As the main interstitial cells in cardiac tissue, cardiac fibroblasts play a central role in the process of post-infarction myocardial fibrosis. In the acute phase of MI, cardiac fibroblasts act as “sentinel cells” and participate in inflammatory activation. A variety of cytokines and growth factors released by injured myocardium, such as transforming growth factor-*β* (TGF-β) and platelet-derived growth factor (PDGF), can activate cardiac fibroblasts and induce their transformation into myofibroblasts (2). Activated cardiac fibroblasts in the acute phase start to synthesize and secrete various extracellular matrix components, including fibronectin and Collagen III, leading to myocardial fibrosis, which further impairs the diastolic and systolic functions of the heart and eventually progresses to heart failure (3). Although the role of fibroblast activation in the acute phase of MI has been partially elucidated, the specific molecular regulatory mechanisms of their activation, such as the key role of signaling pathways in initiating fibroblast activation and how different stimuli precisely regulate fibroblast activation, remain not fully understood and warrant further in-depth investigation.

Recent studies have indicated that lactate levels are significantly elevated in cardiac tissue and serum after MI, and lactate is closely associated with the process of post-infarction myocardial fibrosis (4). Long regarded as the end product of glycolysis and a metabolic waste (5), the biological functions of lactate have been re-recognized in recent years (6). At present, there is no effective therapeutic method to reverse the process of post-infarction cardiac fibrosis.

In recent years, an increasing number of cardiac rehabilitation studies have demonstrated that exercise is an effective intervention for cardiovascular diseases (7–9). Our previous series of studies have confirmed that aerobic exercise (AE) can improve ventricular remodeling caused by MI, enhance cardiac function, increase cardiomyocyte survival rate, and exert a positive effect on ameliorating post-infarction cardiac remodeling (10). AE can promote cardiac angiogenesis, enhance myocardial contractility, improve myocardial energy metabolism, and inhibit myocardial fibrosis (8). Exercise-induced factors play an important mediating role in exercise-induced cardiac growth and protection, among which insulin-like growth factor-1 (IGF1) has attracted extensive attention (11). As a polypeptide growth factor secreted by various tissues under exercise stimulation, IGF1 can reach the heart through the blood circulation, bind to IGF1 receptors on the surface of cardiomyocytes, and activate a series of downstream signaling pathways. It exerts multiple effects including promoting cardiomyocyte survival and proliferation, inhibiting cardiomyocyte apoptosis, and regulating the function of cardiac fibroblasts (12).

This study aimed to investigate how AE affects the activation of cardiac fibroblasts after MI through lactate and thereby modulates post-infarction fibrosis. Through this research, we hope to provide new theoretical basis and ideas for the prevention and treatment of early cardiac fibrosis after myocardial infarction.

## Methods

### Animal experiments

Male C57BL/6 J mice aged 8–10 weeks were purchased from Beijing Huafukang Biotechnology Co., Ltd. The mice were housed in a specific pathogen-free (SPF) environment with standardized conditions: room temperature of 23 ± 2 °C, relative humidity of 50% ± 10%, and a 12 h light/dark cycle. Food and water were available ad libitum. All experimental procedures were performed in accordance with the Guide for the Care and Use of Laboratory Animals published by the National Institutes of Health (NIH Publication No. 85-23, revised 1996) and approved by the Animal Ethics Committee of Tianjin Medical University (Approval No.: TMUaMEC2018037).

### Establishment of MI model

Male C57BL/6 J mice aged 8–10 weeks were used to establish the MI model by permanent ligation of the left anterior descending coronary artery (LAD), with reference to the methods of our research group (13, 14). Mice in the sham operation group underwent thoracotomy only without vascular ligation. The detailed procedures were as follows: mice were anesthetized with isoflurane and placed on a 37 °C heating pad. A small incision was made between the 3rd and 4th intercostals with mosquito forceps, and the heart was extruded from the thoracic cavity. After locating the LAD, ligation was performed with 6–0 silk suture at approximately 3 mm from its origin. Finally, air was extruded from the thoracic cavity, the chest wall was closed, and the skin was sutured.

### Criteria for successful establishment of the MI model

A dual criterion consisting of intraoperative electrocardiographic (ECG) monitoring and direct visual inspection was used to determine the successful establishment of the mouse myocardial infarction (MI) model. Continuous ECG monitoring was performed throughout the procedure, and changes in lead II were recorded. Following permanent ligation of the left anterior descending coronary artery (LAD), an immediate upwardly concave ST-segment elevation that persisted for at least 5 min without regression was regarded as the characteristic electrocardiographic sign of myocardial ischemia, and served as the core electrophysiological indicator for successful MI model establishment (15, 16). Mice that did not exhibit this typical ST-segment change were excluded immediately, indicating an inappropriate ligation site or insufficient occlusion of the LAD.

Simultaneously, macroscopic changes in cardiac color and morphology were closely observed intraoperatively. Immediately after LAD ligation, pallor and softening of the anterior left ventricular wall, accompanied by loss of local myocardial contractility and abolition of synchronous systolic motion, were recognized as key morphological indicators of successful myocardial ischemia. In addition, the absence of severe surgical complications and maintenance of spontaneous respiration were also adopted as essential intraoperative criteria for valid MI model construction (13, 14).

### Exercise intervention

The exercise intervention protocol was performed with reference to previous literature (14, 17). Mice were divided into four groups. The SED-SHAM group underwent sham MI operation after 2 weeks of sedentary housing. The AE-SHAM group underwent sham MI operation after 2 weeks of AE. The SED-MI group underwent MI operation after 2 weeks of sedentary housing. The AE-MI group underwent MI operation after 2 weeks of AE. All mice were housed in standard cages routinely. Mice in the AE-SHAM and AE-MI groups received treadmill training using a treadmill from Beijing Zhongshi Dichuang Technology Co., Ltd. The exercise intensity was set at 50–60% of the maximum oxygen uptake (VO2max), with 49 min of training per day, 5 days a week, for a total of 2 weeks. A 5-day adaptive training was conducted before the formal exercise intervention. A 5-min warm-up at 40% of VO2max was performed before each training session.

### Cell culture and treatment

NIH 3 T3 mouse embryonic fibroblasts were purchased from Wuhan Zishan Biotechnology Co., Ltd. and cultured in DMEM medium (Gibco, Carlsbad, CA, United States) supplemented with 50 μg/mL penicillin–streptomycin (Gibco) and 10% fetal bovine serum (FBS, Gibco).

To investigate the effects of different stimuli on fibroblast activation, fibroblasts were treated with different concentrations of lactate (0, 1 mM, 2 mM, 5 mM; L-sodium lactate, Sigma-Aldrich), TGF-β1 (0, 5 ng/mL, 10 ng/mL, 20 ng/mL; recombinant human transforming growth factor-β1, Solarbio), and IGF1 (100 ng/mL, recombinant insulin-like growth factor 1, simulating the *in vitro* effect of exercise) for 48 h. Cell morphology was observed under a microscope, and proteins were extracted for Western blot analysis.

### Detection of exercise capacity

Mice were placed on a treadmill with a 15° incline at an initial speed of 6 m/min, and the speed was increased by 3 m/min every 3 min until exhaustion. The total exercise distance was defined as exercise capacity (14, 18). The criterion for exhaustion was that mice could not keep up with the treadmill speed.

### Echocardiographic detection

Echocardiography was performed using a Visual Sonics Vevo 770 high-resolution imaging system equipped with a 15 MHz linear array probe (Visual Sonics, Inc., Toronto, Canada). Mice were anesthetized with isoflurane, and two-dimensional short-axis parasternal imaging was performed. M-mode echocardiography was measured at the level of the papillary muscle in the mid-ventricle. Left ventricular ejection fraction (LVEF) and left ventricular fractional shortening (LVFS) were calculated using the built-in software of the ultrasound system.

### Western blot analysis

Myocardial tissue at the infarct border zone or fibroblasts were lysed with protein lysis buffer, and protein concentration was determined using a BCA kit (Thermo, 23,225). An equal amount of 50 μg protein per lane was loaded for sodium dodecyl sulfate-polyacrylamide gel electrophoresis (SDS-PAGE). Proteins were transferred to nitrocellulose membranes at a constant voltage of 80 V, and the membranes were blocked with 5% non-fat milk at room temperature for 2 h. The membranes were incubated overnight at 4 °C with primary antibodies against Collagen I (COL I, 1:2000 dilution; 14,695-1-AP, Proteintech), *α*-SMA (1:2000 dilution; GB111364, Servicebio), and GAPDH (1:2000 dilution; 10,494-1-AP, Proteintech). Subsequently, the membranes were incubated with horseradish peroxidase (HRP)-conjugated secondary antibodies (1:2000 dilution; Cell Signaling Technology) at room temperature for 2 h. Protein bands were visualized by enhanced chemiluminescence using a gel imaging system.

### Quantitative real-time PCR detection (qRT-PCR)

The mRNA levels of *Col1a2* and *Acta2* genes were detected by qRT-PCR with reference to previous literature (19). Briefly, total RNA was extracted and reverse-transcribed into cDNA, followed by real-time PCR amplification using SYBR Green Master Mix. The relative quantification of mRNA levels was performed using the 2 − ΔΔCT method, normalized to ACTB or 18sRNA as the internal reference gene. The primer sequences used in this experiment are detailed in Supplementary Table S1.

### Lactate level detection

Mouse serum was separated from the supernatant of clotted whole blood by centrifugation at 2500 g for 10 min at 4 °C (4). Lactate levels in cardiac tissue and serum were determined in accordance with the instructions of the L-lactic acid (L-LA) assay kit (AKAC001-1 M, Boxbio, China).

### Collection of mouse serum

The chest and abdomen of mice to be detected were depilated with depilatory cream in advance. Mice were anesthetized with isoflurane and placed in a supine position on an operating board. The skin in the middle of the abdomen was clamped with forceps, a small incision was made and extended upward to the chest along both sides of the abdomen. Subcutaneous tissue was bluntly dissected, the diaphragm was incised to expose the beating heart, and approximately 900 μL of blood was slowly drawn from the right ventricle with a 1 mL syringe. The blood was slowly injected into a coagulation tube and allowed to stand for 2 h until pale yellow serum appeared above the coagulated blood clot. The coagulation tube was then centrifuged at 2500 g for 10 min at 4 °C, and the serum was aspirated into a sterile centrifuge tube with a sterile pipette (4).

### Metabolomics detection

Mice in the SED-MI group underwent MI operation under sedentary conditions, while mice in the SED-MI + LAC group were intraperitoneally injected with lactate (0.5 g/kg) once a day for 7 consecutive days after MI operation under sedentary conditions (4). Serum samples from the SED-MI and SED-MI + LAC groups were pretreated for metabolite extraction. 100 μL of pre-cooled extraction solution (methanol: acetonitrile: water = 2:2:1, v/v/v) containing isotopically labeled internal standards at −40 °C was added to the samples. The mixture was vortexed for 30 s, sonicated for 10 min in an ice-water bath, and incubated at −40 °C for 1 h. The samples were centrifuged at 12000 rpm for 15 min at 4 °C, and the supernatant was collected into injection vials for instrumental analysis. Chromatographic separation of target compounds was performed using a Vanquish ultra-performance liquid chromatography (UPLC) system (Thermo Fisher Scientific) with a Waters ACQUITY UPLC BEH Amide column (2.1 mm × 50 mm, 1.7 μm). Mass spectrometry data (MS1 and MS2) were acquired using an Orbitrap Exploris 120 mass spectrometer controlled by Xcalibur software (Version 4.4, Thermo). Raw data were processed using ProteoWizard software (V3.0.24054) for metabolite identification based on the BiotreeDB database (V3.0).

### Statistical analysis

Data processing and statistical analysis were performed using SPSS 26.0 software. The normality of quantitative data was verified by the Shapiro–Wilk test. Data with normal distribution were presented as mean ± standard error of the mean (Mean ± SEM). Student’s t-test was used for comparison between two groups. One-way analysis of variance (ANOVA) was applied for comparisons among three or more groups, and two-way ANOVA was used for two-factor analysis. *p* value < 0.05 was considered statistically significant.

## Results

### Lactate induces fibroblast activation

**Figure 1:**
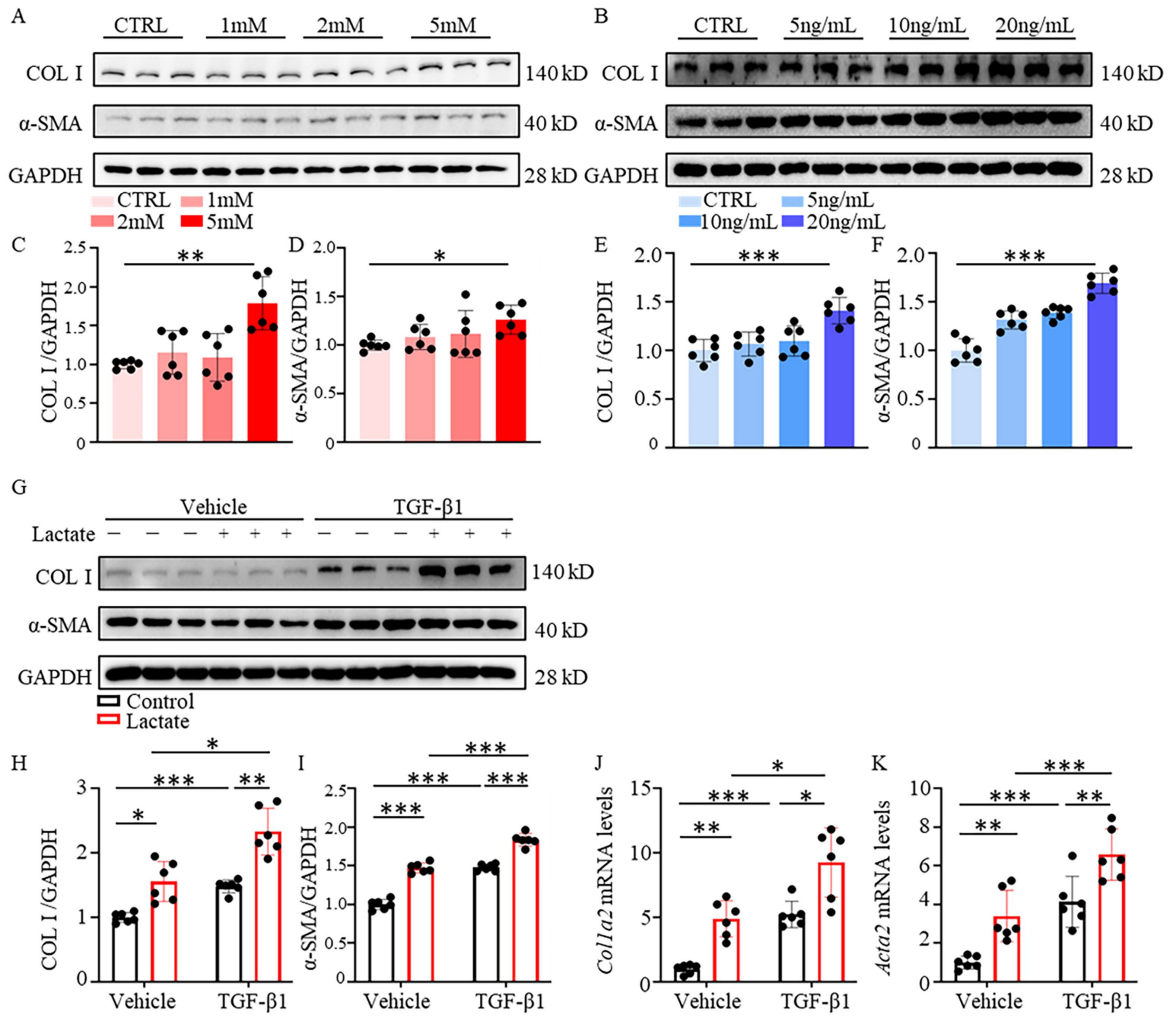
Lactate induces NIH 3 T3 cells activation. **(A)** Expression of COL I and *α*-SMA in NIH 3 T3 cells stimulated with lactate at different concentrations (CTRL, 1 mM, 2 mM, 5 mM). **(B)** Expression of COL I and α-SMA in NIH 3 T3 cells stimulated with TGF-*β*1 at different concentrations (CTRL, 5 ng/mL, 10 ng/mL, 20 ng/mL). **(C,D)** Quantitative analysis of COL I and α-SMA protein levels in NIH 3 T3 cells stimulated with lactate by Western blot (*n* = 6). **(E,F)** Quantitative analysis of COL I and α-SMA protein levels in NIH 3 T3 cells stimulated with TGF-β1 by Western blot (*n* = 6). **(G)** Expression of COL I and α-SMA in NIH 3 T3 cells stimulated with 5 mM lactate and 20 ng/mL TGF-β1. **(H,I)** Quantitative analysis of COL I and α-SMA protein levels in NIH 3 T3 cells stimulated with 5 mM lactate and 20 ng/mL TGF-β1 by Western blot (*n* = 6). **(J,K)** mRNA expression levels of *Col1a2* and *Acta2* in NIH 3 T3 cells stimulated with 5 mM lactate and 20 ng/mL TGF-β1 determined by qRT-PCR (*n* = 6). All data are presented as Mean ± SEM. ^*^p < 0.05, ^**^p < 0.01, ^***^p < 0.001.

The activation of cardiac fibroblasts into myofibroblasts is a core link in the occurrence of post-infarction cardiac fibrosis. However, whether lactate, a metabolite abnormally accumulated in myocardial tissue after MI, directly regulates fibroblast activation remains unclear. To explore the direct effect of lactate on cardiac fibroblast activation, *in vitro* experiments were conducted on NIH 3 T3 fibroblasts. Fibroblasts were first stimulated with different concentrations of lactate (0, 1 mM, 2 mM, 5 mM), and the results showed that the expression of fibrosis-related markers (COL I and *α*-SMA) was significantly increased with the increase of lactate concentration, especially in the 5 mM lactate stimulation group(*P*_COL I_ = 0.008, *P*_α-SMA_ = 0.025; Figures 1A,C,D). Meanwhile, fibroblasts were stimulated with TGF-β1, a classic fibrosis-inducing factor, at different concentrations (0, 5 ng/mL, 10 ng/mL, 20 ng/mL). The most obvious fibroblast activation was observed in the 20 ng/mL TGF-β1 group, with significantly elevated expression of COL I and *α*-SMA (*P*_COL I_ < 0.001, *P*_α-SMA_ < 0.001; Figures 1B,E,F). Co-stimulation with 5 mM lactate and 20 ng/mL TGF-β1 resulted in the most significant fibroblast activation, and the expression of COL I and α-SMA was significantly higher than that in the single lactate or single TGF-β1 group(*P*_COL I_ = 0.012, *P*_α-SMA_ < 0.001). The results of Western blot (Figures 1G,I) were consistent with those of qPCR (Figures 1J,K). All the above results indicated that lactate can directly induce the activation of cardiac fibroblasts *in vitro*, and has a synergistic regulatory effect with TGF-β1, a classic fibrosis pathway factor, to jointly promote the transformation of fibroblasts into myofibroblasts.

### Elevated lactate level induces arachidonoyl carnitine accumulation and impairs cardiac function after MI

**Figure 2:**
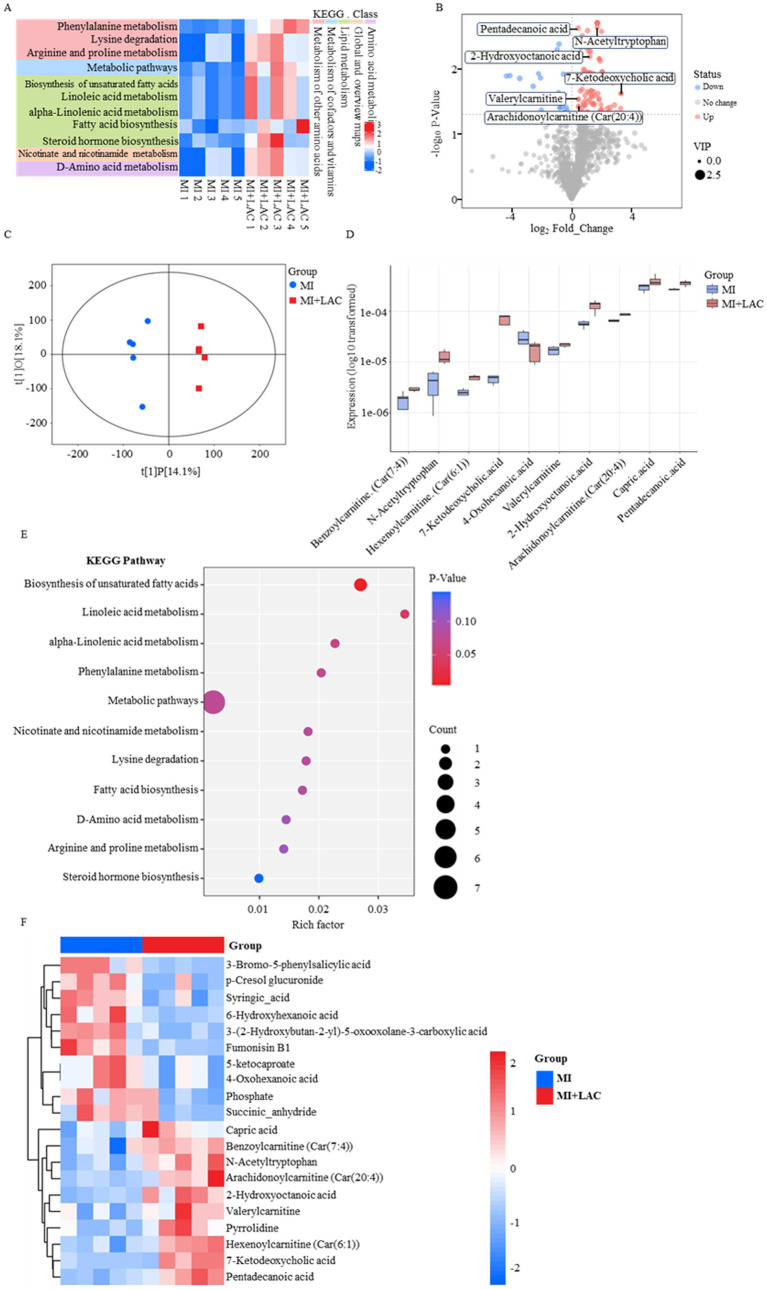
Metabolomic profiling of serum from SED-MI and SED-MI + LAC mice. **(A)** KEGG pathway enrichment heatmap of differential metabolites. **(B)** Volcano plot of differential metabolites. **(C)** OPLS-DA score plot for SED-MI and SED-MI + LAC groups. **(D)** Box plot of top differential metabolite expression. **(E)** Bubble plot of KEGG pathway enrichment analysis. **(F)** Hierarchical clustering heatmap of differential metabolites.

After MI, myocardial tissue undergoes a significant shift in energy metabolism from aerobic oxidation to glycolysis due to ischemia and hypoxia (20), and lactate is a key end product of the glycolytic pathway (21). We hypothesized that metabolic disorders occur after MI, and abnormal lactate accumulation impairs post-infarction cardiac function. To verify this hypothesis, metabolomics analysis was performed on the serum of mice in the SED-MI and SED-MI + LAC groups (Figures 2A–F). The results showed that the level of arachidonoyl carnitine was significantly up-regulated in the SED-MI + LAC group (Figures 2A,B), and KEGG analysis revealed obvious metabolic pathway disorders (Figures 2A,E). Arachidonoyl carnitine is a derivative formed by the esterification of arachidonic acid and carnitine (22). Previous studies have shown that arachidonic acid mediates tissue fibrosis by activating the release of inflammatory factors and promoting extracellular matrix deposition (23, 24), and the down-regulation of arachidonic acid metabolism exerts an anti-pulmonary fibrosis effect by inhibiting the TGF-β1/Smad pathway (25). Combined with the metabolomics results, it can be inferred that elevated lactate levels after MI lead to abnormal accumulation of arachidonoyl carnitine, which suggests that arachidonoyl carnitine may act as a metabolic signal involved in the regulation of fibrosis-related pathways.

### IGF1 inhibits lactate-induced fibroblast activation

**Figure 3:**
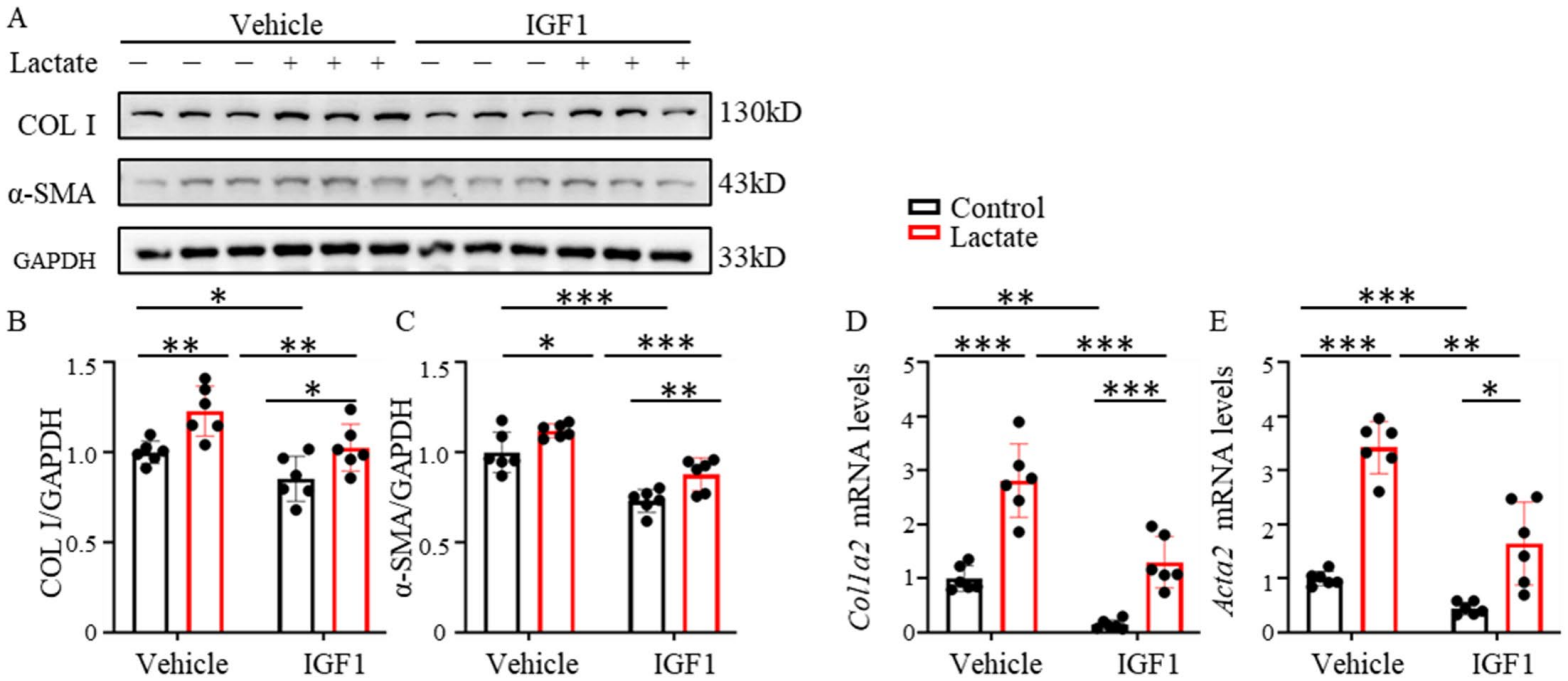
IGF1 inhibits lactate-induced NIH 3 T3 activation. **(A)** Expression of COL I and α-SMA in NIH 3 T3 stimulated with 5 mM lactate and 100 ng/mL IGF1. **(B,C)** Quantitative analysis of COL I and α-SMA protein levels in NIH 3 T3 stimulated with 5 mM lactate and 100 ng/mL IGF1 by Western blot (*n* = 6). **(D,E)** mRNA expression levels of *Col1a2* and *Acta2* in NIH 3 T3 stimulated with 5 mM lactate and 100 ng/mL IGF1 determined by qRT-PCR (*n* = 6). Data are expressed as Mean ± SEM, ^*^p < 0.05, ^**^p < 0.01, ^***^p < 0.001.

Based on the research background that AE can improve post-infarction cardiac function (13), we hypothesized that AE may inhibit the pro-fibrotic effect of lactate by regulating specific factors. As a classic exercise-induced factor, IGF1 is secreted by various tissues under exercise stimulation and participates in cardiac protection, but whether it regulates lactate-mediated cardiac fibroblast activation remains unclear. To explore the effect of exercise-induced factors on the pro-fibrotic effect of lactate, IGF1 was used to simulate the *in vitro* effect of AE to stimulate fibroblasts (13). Fibroblasts were co-stimulated with lactate and IGF1, and the results of Western blot showed that the expression of COL I and *α*-SMA were significantly decreased compared with the lactate alone group (*p* < 0.05). The results of Western blot (Figures 3A–C) were consistent with those of qPCR (Figures 3D–E). The above results indicated that IGF1 can inhibit lactate-induced fibroblast activation.

### AE reduces lactate accumulation and ameliorates post-MI cardiac function

**Figure 4:**
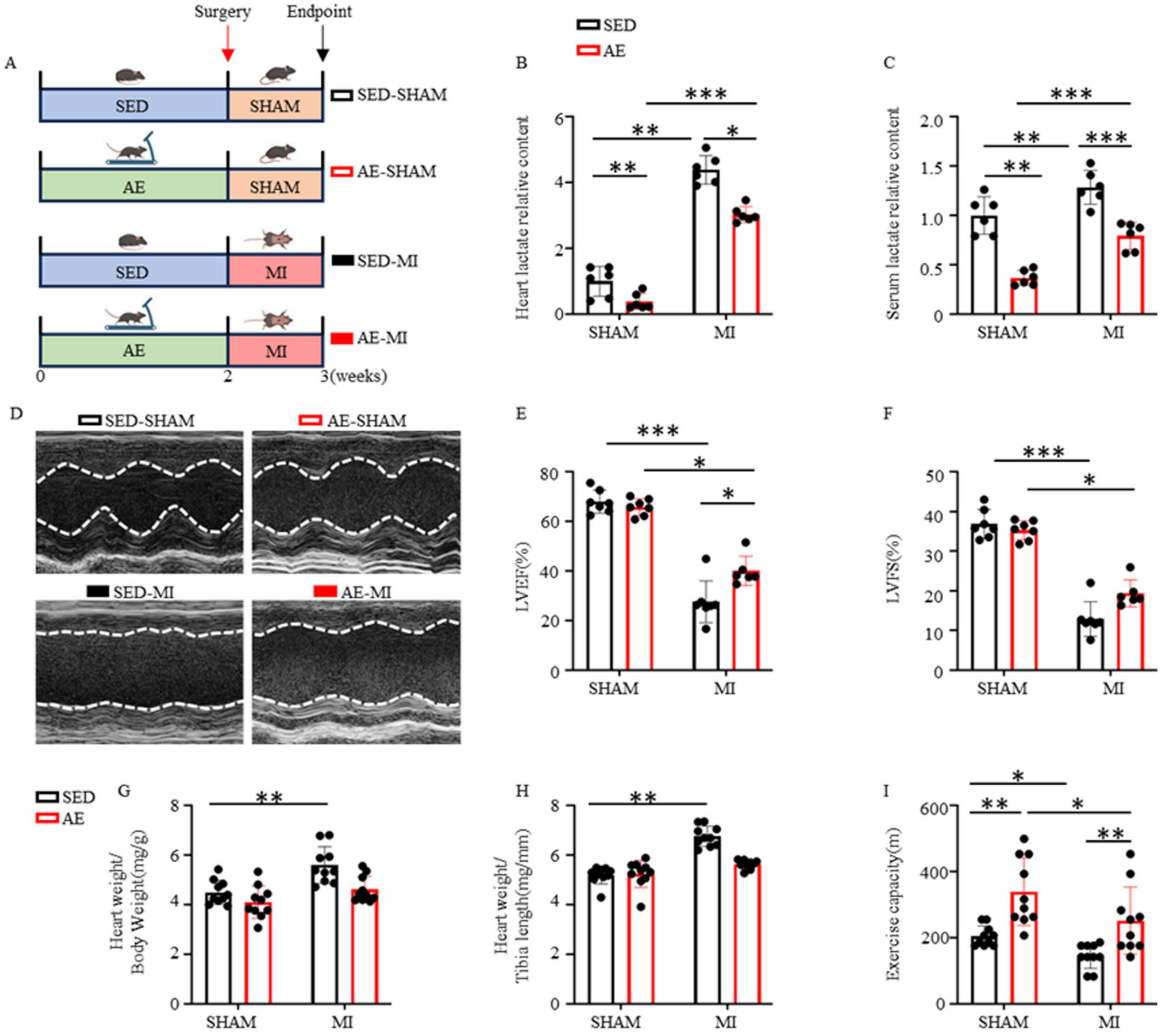
AE reduces lactate accumulation and ameliorates post-MI cardiac function. **(A)** Schematic illustration of the experimental animal grouping design. **(B)** Cardiac tissue lactate levels in each group (*n* = 6). **(C)** Serum lactate levels in each group (*n* = 6). **(D)** Representative echocardiographic images from each experimental group. **(E)** Comparison of LVEF across groups (*n* = 6–7). **(F)** Comparison of LVFS across groups (*n* = 6–7). **(G)** Heart weight to body weight ratio of animals in each group (*n* = 10). **(H)** Heart weight to tibia length ratio of animals in each group (*n* = 10). **(I)** Exercise capacity ratio of animals in each group (n = 10). Data are expressed as Mean ± SEM, ^ns^*P* > 0.05, ^*^p < 0.05, ^**^p < 0.01, ^***^p < 0.001.

Previous studies have shown that lactate levels are significantly elevated in myocardial tissue and peripheral serum of mice after MI, and the abnormal elevation of lactate levels can promote the occurrence and development of post-infarction myocardial fibrosis by inducing the endothelial-mesenchymal transition of cardiac endothelial cells (4). To clarify the potential correlation between lactate levels and cardiac function, four animal models were designed in this study (SED-SHAM, AE-SHAM, SED-MI and AE-MI group) (Figure 4A), and lactate levels in myocardial tissue and peripheral serum of mice in each group were systematically detected and compared. The results showed that lactate levels in cardiac tissue and serum of the SED-MI group were significantly higher than those of the SED-SHAM group, which was consistent with previous studies. Compared with the SED-SHAM group, the SED-MI group showed significantly elevated levels of both heart lactate and serum lactate (*P*_heart lactate_ < 0.001, *P*_serum lactate_ = 0.004). Compared with the SED-SHAM group, the AE-SHAM group exhibited significantly decreased levels of cardiac lactate and serum lactate (*P*_heart lactate_ = 0.007, *P*_serum lactate_ < 0.001). Compared with the AE-SHAM group, the AE-MI group presented significantly increased levels of cardiac lactate and serum lactate (*p* < 0.001). Compared with the SED-MI group, the AE-MI group displayed significantly reduced levels of cardiac lactate and serum lactate (*p* < 0.001; Figures 4B,C).

In addition, compared with the SED-SHAM group, the AE-SHAM group showed significantly increased exercise capacity, while no significant differences were observed in the heart weight/body weight ratio or heart weight/tibial length ratio (*P*_heart weight/body weight_ = 0.158, *P*_heart weight/tibial length_ = 0.991, *P*_exercise capacity_ = 0.010; Figures 4G–I).

Compared with the SED-SHAM group, the SED-MI group exhibited significantly elevated heart weight/body weight and heart weight/tibial length ratios, along with markedly decreased exercise capacity (*P*_heart weight/body weight_ < 0.001, *P*_heart weight/tibial length_ < 0.001, *P*_exercise capacity_ = 0.005). Compared with the SED-MI group, the AE-MI group showed significantly reduced heart weight/body weight and heart weight/tibial length ratios, as well as significantly increased exercise capacity (*P*_heart weight/body weight_ = 0.001, *P*_heart weight/tibial length_ < 0.001, *P*_exercise capacity_ = 0.039). Compared with the AE-SHAM group, no significant differences were detected in the heart weight/body weight ratio, heart weight/tibial length ratio, or exercise capacity in the AE-MI group (*P*_heart weight/body weight_ = 0.068, *P*_heart weight/tibial length_ = 0.206, *P*_exercise capacity_ = 0.260; Figures 4G–I). The above data indicated that AE and MI result in different lactate levels in cardiac tissue and serum, which further affect cardiac function.

To clarify the changes in cardiac function of mice in each group, echocardiography was performed (Figure 4D). The results demonstrated that, compared with the SED-SHAM group, the SED-MI group exhibited significantly decreased LVEF and LVFS (*P*_LVEF_ < 0.001, *P*_LVFS_ < 0.001). Compared with the AE-SHAM group, the AE-MI group showed markedly reduced LVEF and LVFS (*P*_LVEF_ < 0.001, *P*_LVFS_ < 0.001). Compared with the SED-MI group, the AE-MI group presented significantly increased LVEF and LVFS (*P*_LVEF_ = 0.043, *P*_LVFS_ = 0.003). No significant differences in LVEF or LVFS were observed between the AE-SHAM group and the SED-SHAM group (*P*_LVEF_ = 0.754, *P*_LVFS_ = 0.420; Figures 4E,F). These results indicated that cardiac function differs between AE and post-infarction states, and cardiac function after MI with AE is better than that after MI without AE, which may be related to the decrease of lactate levels in cardiac tissue and serum after AE.

### Correlation between lactate levels and cardiac function in mice

**Figure 5:**
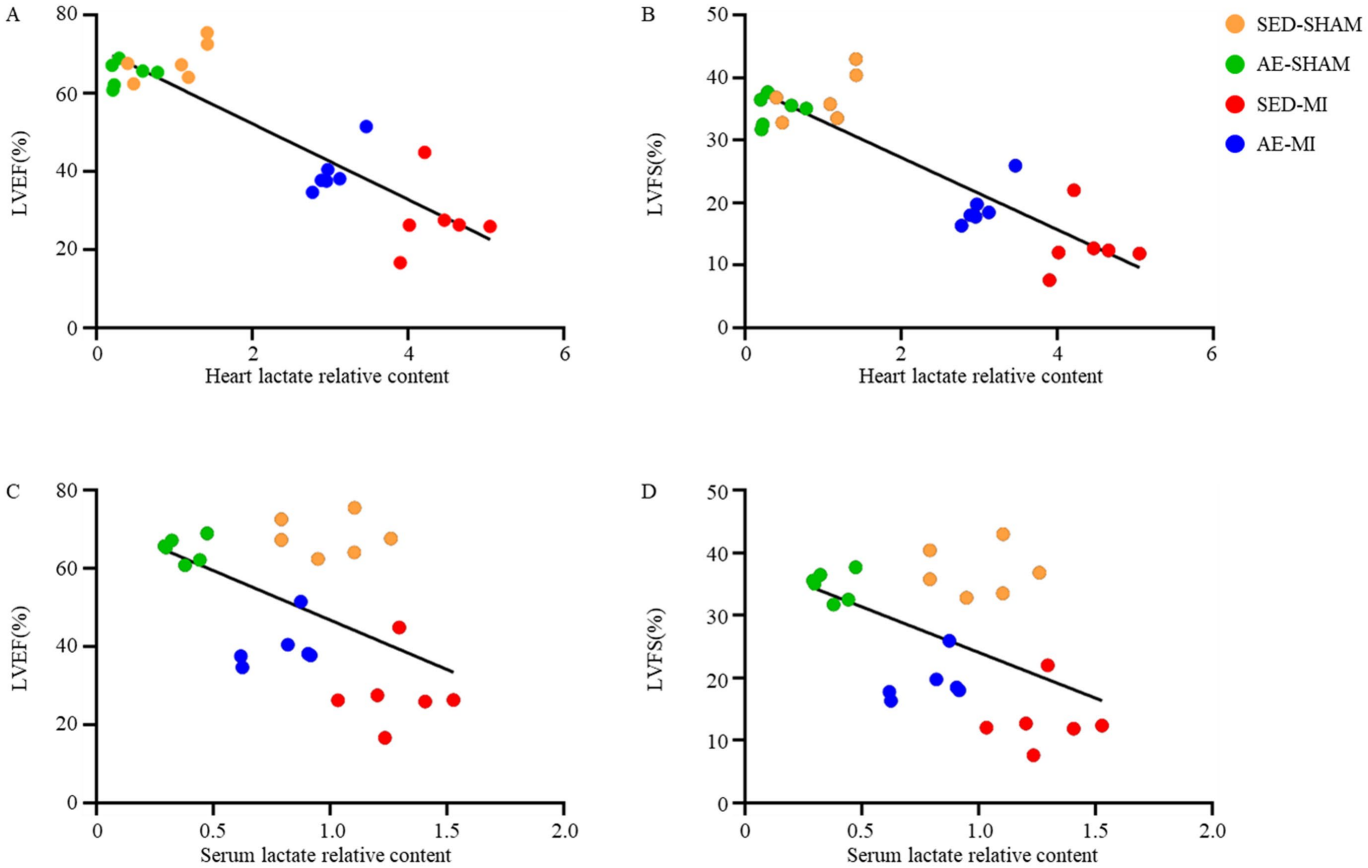
Correlation between lactate levels and cardiac function in mice. **(A)** Correlation between cardiac lactate and LVEF. **(B)** Correlation between cardiac lactate and LVFS. **(C)** Correlation between serum lactate and LVEF. **(D)** Correlation between serum lactate and LVFS.

To clarify the relationship between lactate levels and post-MI cardiac dysfunction, we performed Pearson correlation analysis and linear regression analysis on data, which were divided into four experimental groups (SED-SHAM, AE-SHAM, SED-MI and AE-MI group). The associations between lactate levels and cardiac function indices were further visualized using scatter plots (Figure 5).

As shown in Figure 5, myocardial lactate relative content exhibited strong negative correlations with both LVEF and LVFS. Serum lactate relative content also correlated negatively with LVEF and LVFS, albeit with weaker strength compared with myocardial lactate. Myocardial lactate levels showed a very strong negative correlation with LVEF (*r* = −0.886, *p* < 0.001) and LVFS (*r* = −0.880, *p* < 0.001), indicating a robust inverse relationship independent of group assignment (Supplementary Table S2). Serum lactate levels showed a moderate negative correlation with LVEF (*r* = −0.512, *p* = 0.011) and LVFS (*r* = −0.493, *p* = 0.014), suggesting that serum lactate reflects cardiac functional status, while myocardial lactate may act as a more direct mediator of dysfunction (Supplementary Table S2).

Sham-operated mice (SED-SHAM and AE-SHAM group) displayed low myocardial lactate levels and preserved LVEF/LVFS, while MI mice (SED-MI and AE-MI group) showed elevated myocardial lactate accompanied by markedly reduced cardiac function. Notably, the AE-MI group had lower myocardial lactate and milder cardiac dysfunction compared with the SED-MI group, further confirming the negative association. Sham-operated mice maintained low serum lactate and normal cardiac function, whereas MI mice exhibited elevated serum lactate and impaired LVEF/LVFS. Consistent with myocardial lactate findings, the AE-MI group had lower serum lactate and better cardiac function than the SED-MI group.

Linear regression analysis revealed that myocardial lactate explained 78.4% of the variance in LVEF and 77.4% of the variance in LVFS (*p* < 0.001). Linear regression analysis demonstrated that serum lactate explained 26.2% of the variance in LVEF and 24.3% of the variance in LVFS (both *p* < 0.05). The 95% confidence intervals for the *β* coefficients were consistently negative, confirming the significant negative predictive value of myocardial lactate and serum lactate for cardiac function (Supplementary Table S3).

## Discussion

AE is a commonly used therapeutic method for cardiac rehabilitation in cardiovascular diseases (26), but its specific mechanism of action remains not fully understood. Myocardial fibrosis after MI is a core pathological event in the process of ventricular remodeling (15, 27), and the early activation of cardiac fibroblasts into myofibroblasts is considered to be the core driving link for the initiation and progression of fibrosis (28). However, the sustained activation of repair pathways induces the development of fibrosis in the infarct border zone, eventually leading to heart failure and even death. Therefore, exploring the specific mechanism of early cardiac fibroblast activation after MI and how AE affects this process is of irreplaceable value for elucidating the regulatory law of post-infarction fibrosis and developing targeted intervention strategies. Through *in vitro* and *in vivo* experiments, this study systematically investigated the role of different lactate levels in the process of early cardiac fibrosis after MI and the regulatory effect of AE. The results showed that elevated lactate levels after MI promote fibroblast activation, while AE reduces lactate levels, thereby inhibiting cardiac fibroblast activation and improving cardiac function.

Recent studies have found that lactate can act as a substrate for lactylation modification and participate in the regulation of a variety of important biological functions (29–31). This study found that lactate levels in myocardial tissue and serum were significantly elevated after MI, while they were decreased after AE preconditioning. Meanwhile, cardiac function was impaired after MI, while it was improved after AE preconditioning, and the cardiac function of mice with MI after exercise preconditioning was better than that of mice with MI alone. Studies have shown that metabolic disorders in the myocardial microenvironment after MI are one of the key inducers of fibrosis (32). Under normal physiological conditions, cardiomyocytes mainly produce energy through aerobic metabolism, which requires sufficient oxygen and results in little lactate production (33). However, after MI, cardiomyocytes cannot perform effective aerobic metabolism due to ischemia and hypoxia and switch to anaerobic glycolysis for energy supply (34). Lactate, the end product of anaerobic glycolysis, accumulates in the myocardium in large quantities and is released into the blood through lactate transporters on the cell membrane, leading to elevated systemic lactate levels (35). AE can enhance myocardial contractility and cardiac output, improve tissue perfusion and oxygen supply (10). At the same time, aerobic exercise activates systemic metabolic regulation. Specifically, skeletal muscle actively takes up lactate from the bloodstream, utilizing it as a substrate for aerobic metabolism to directly meet energy demands. Meanwhile, the liver and kidneys enhance lactate metabolic capacity, for instance by converting lactate to glucose via gluconeogenesis to expedite lactate clearance. Additionally, aerobic exercise improves mitochondrial function and enhances the activity of aerobic metabolic enzymes. This enables more efficient breakdown of glucose and fatty acids through aerobic pathways, thereby effectively reducing lactate production (36).

The activation of cardiac fibroblasts after cardiac injury is triggered by a variety of signals, including cytokines, inflammatory factors and growth factors released by injured myocardium and immune cells, but the role of these extracellular fibrotic signal transductions in cardiac fibroblasts remains unclear (37). Studies have shown that lactate can activate latent TGF-*β* to increase collagen synthesis and enhance glycolysis, thereby promoting the activation of pulmonary and renal fibroblasts as well as hepatic stellate cells (38, 39). Previous studies on heart diseases have mostly focused on cardiomyocytes, endothelial cells and inflammatory cells, while this study for the first time focused on cardiac fibroblasts and carried out a series of studies on the effect of AE preconditioning on the activation of cardiac fibroblasts in the early stage after MI. To determine whether lactate acts as a key metabolic mediator in the activation of cardiac fibroblasts, the present study first performed *in vitro* experiments using NIH 3 T3. The results confirmed that lactate significantly promoted fibroblast activation, whereas the exercise-induced factor IGF1 markedly inhibited this effect. In future studies, we will further verify these findings using neonatal rat cardiac fibroblasts (NRCFs) to better recapitulate the pathophysiological features of the cardiac microenvironment after myocardial infarction. These results were highly consistent with the *in vivo* experimental results that AE reduces lactate accumulation and ameliorates post-MI cardiac function. It not only provides in vitro experimental evidence for the pathological mechanism of myocardial fibrosis mediated by abnormal lactate metabolism after MI (4), but also further verifies the core hypothesis that AE exerts a cardioprotective effect after MI by regulating lactate metabolism.

Previous studies have confirmed the existence of metabolic disorders after MI (40, 41). Metabolomic results showed that lactate intervention significantly induced abnormal accumulation of arachidonoyl carnitine, accompanied by disorders in lipid metabolic pathways. As a key intermediate in arachidonic acid metabolism, arachidonoyl carnitine can promote inflammation and fibrosis by releasing free arachidonic acid to activate the NF-κB and TGF-β1/Smad pathways. Meanwhile, it can cause lipotoxicity by inhibiting the AMPK pathway, further amplifying fibrotic responses (21–24). The findings of the present study suggest that lactate drives fibroblast activation at the metabolic level not merely through extracellular signal stimulation, but also by remodeling lipid metabolism and inducing arachidonoyl carnitine accumulation. Although direct functional verification such as knockdown, overexpression, or pharmacological intervention of arachidonoyl carnitine was not performed in this study, arachidonoyl carnitine has been validated as a functional metabolic mediator rather than a passive byproduct in multi-organ fibrosis (22). This study for the first time associated arachidonoyl carnitine with lactate metabolism, suggesting that lactate accumulation after MI may accelerate the process of fibrosis by remodeling lipid metabolism. In addition, studies have reported that lactate promotes endothelial-mesenchymal transition through the TGF-β1-Smad pathway under hypoxic conditions, thereby promoting the occurrence of post-infarction fibrosis (4). Future studies may further verify the specific mechanisms of this metabolic axis by targeting acyltransferases or carnitine transporters.

Although this study initially clarified the role and correlation of lactate and AE in post-infarction cardiac fibrosis, there are still many limitations without in-depth mechanism exploration, mainly reflected in the following aspects. First, we only confirmed that lactate can induce the accumulation of arachidonoyl carnitine, but the specific regulatory relationship between them remains unclear. Whether lactate mediates the synthesis and accumulation of arachidonoyl carnitine by regulating key molecules such as acyltransferases or carnitine transporters needs further verification. Second, it was confirmed that IGF1 can inhibit lactate-induced fibroblast activation, but the specific signaling pathway of IGF1 regulating lactate metabolism or fibrosis has not been elucidated. Third, the specific molecular mechanism of lactate regulating cardiac fibroblast activation has not been explored, and whether lactate regulates the expression of fibrosis-related proteins or genes through lactylation modification remains to be verified.

As a core strategy of cardiac rehabilitation, the underlying mechanisms of aerobic exercise remain incompletely elucidated. The present study confirmed that aerobic exercise preconditioning markedly reduced lactate levels in myocardial tissue and serum of mice with myocardial infarction, along with significant improvements in LVEF and LVFS. To verify the causal relationship between lactate and cardiac function, individual-level Pearson correlation analysis and linear regression analysis were conducted in four groups. The results demonstrated that myocardial lactate was strongly negatively correlated with LVEF and LVFS, while serum lactate was moderately negatively correlated with these functional parameters. Regression analysis further revealed that lactate levels significantly accounted for the variance in cardiac function, excluding a simple coincidental association. Furthermore, aerobic exercise established a sequential interventional relationship characterized by reducing lactate accumulation, subsequently alleviating myocardial fibrosis, and ultimately improving cardiac function. This causal chain provides further evidence supporting a direct causal link between lactate accumulation and cardiac dysfunction, rather than a mere incidental association derived from group differences.

Although the present study has preliminarily clarified the roles and intrinsic relationships of lactate and aerobic exercise in post-infarction cardiac fibrosis, several limitations still exist. First, this study did not use specific lactate inhibitors or lactate transporter blockade experiments to directly verify the specificity of lactate in regulating fibroblast activation. Second, this study was only performed in NIH 3 T3 embryonic fibroblasts and has not been repeated and verified in primary cardiac fibroblasts. Third, this study did not deeply explore the specific downstream signaling pathways by which arachidonoyl carnitine mediates fibrosis. Fourth, this study did not perform rescue experiments such as IGF1 knockout or knockdown to verify the specificity of IGF1 in inhibiting lactate-induced effects. Fifth, this study did not directly detect the key molecular mechanisms underlying lactate reduction by aerobic exercise, including mitochondrial function and the expression of lactate transporters (MCTs). Sixth, this study only used male mice and did not evaluate the influence of gender differences on lactate metabolism, fibrosis, and exercise-induced effects (42). These limitations have been fully addressed in this article and also point out directions for further in-depth research.

To address the above limitations, future studies will be improved in several aspects. First, the specificity of lactate in regulating cardiac fibroblast activation will be clarified using pharmacological tools including lactate inhibitors and transporter blockers. Second, key experiments will be repeated in primary cardiac fibroblasts to improve the physiological relevance and reliability of the findings. Third, the downstream signaling pathways of arachidonoyl carnitine, including NF-κB, TGF-β1/Smad, and AMPK, will be thoroughly investigated to elucidate the specific molecular mechanisms underlying fibrosis. Fourth, rescue experiments such as IGF1 knockdown, gene silencing, and receptor blockade will be used to verify the specificity and key pathways of IGF1. Fifth, mitochondrial respiratory function, aerobic metabolic enzyme activities, and the expression of MCT1/MCT4 will be detected to clarify the core molecular mechanisms by which aerobic exercise reduces lactate levels. Sixth, female mice will be included for parallel experiments to evaluate gender differences in lactate metabolism, myocardial fibrosis, and the cardioprotective effects of exercise. On this basis, clinical samples from patients with myocardial infarction will be further collected to verify the correlations between lactate metabolic parameters, cardiac function, and fibrosis, so as to promote the translation of basic research findings into clinical cardiac rehabilitation.

In summary, this study initially confirmed through *in vitro* and *in vivo* experiments that abnormal lactate accumulation after MI can directly promote the activation of cardiac fibroblasts and mediate metabolic disorders by inducing the accumulation of arachidonoyl carnitine, thereby aggravating cardiac function injury. AE can inhibit lactate-induced fibroblast activation by reducing lactate accumulation and up-regulating IGF1 expression, and ultimately improve cardiac function after myocardial infarction. This study for the first time associated lactate, arachidonoyl carnitine metabolism with post-infarction cardiac dysfunction, and clarified the cardioprotective effect of AE by regulating lactate metabolism, providing a new theoretical basis for the prevention and treatment of post-infarction fibrosis by targeting metabolic pathways. In-depth mechanism research and clinical transformation verification in the future will further promote the application of lactate metabolic targets in the prevention and treatment of post-infarction fibrosis, and provide new ideas for the combination of clinical cardiac rehabilitation and anti-fibrotic therapy.

## Conclusion

This study identifies lactate as a critical pathogenic mediator of cardiac dysfunction following MI, with its accumulation contributing to the progression of post-infarction cardiac impairment. AE exerts a cardioprotective effect by reducing lactate levels in cardiac tissue and serum, thereby mitigating the adverse consequences of lactate accumulation on cardiac function. The exercise-induced factor IGF1 further supplements this protective effect by inhibiting lactate-mediated cardiac fibroblast activation, while lactate-induced arachidonoyl carnitine accumulation suggests a potential metabolic regulatory pathway underlying post-MI cardiac dysfunction. Collectively, these findings highlight lactate metabolism as a promising therapeutic target for post-MI cardiac dysfunction and reinforce the clinical significance of AE in cardiac rehabilitation, providing new theoretical support for the development of targeted interventions to improve outcomes in MI patients.

## Data Availability

The original contributions presented in the study are included in the article/Supplementary material, further inquiries can be directed to the corresponding author/s.
