# Supplementary material for: Aerobic exercise reduces lactate accumulation and improves cardiac function after myocardial infarction
Source: Front Med (Lausanne). 2026 Apr 20;13:1816790. doi: 10.3389/fmed.2026.1816790 (PMC13135995; doi:10.3389/fmed.2026.1816790)
Supplement: Supplementary file 1 [file Supplementary_file_1.docx]

**Table S1. Primers for quantitative real-time PCR**

| Gene | Forward primer（5’→3’） | Reverse primer（5’→3’） |
| --- | --- | --- |
| Col1a2 | GTAACTTCGTGCCTAGCAACA | CCTTTGTCAGAATACTGAGCAGC |
| Acta2 | GTCCCAGACATCAGGGAGTAA | TCGGATACTTCAGCGTCAGGA |
| Gapdh | AGGTCGGTGTGAACGGATTTG | TGTAGACCATGTAGTTGAGGTCA |

**Table S2. Pearson correlation coefficients between lactate levels and cardiac function parameters**

| **Independent Variable** | **Dependent Variable** | **Pearson Correlation Coefficient (r)** | ***P*-Value** |
| --- | --- | --- | --- |
| Heart lactate | LVEF (%) | -0.886 | <0.001 |
| Hear lactate | LVFS (%) | -0.880 | <0.001 |
| Serum lactate | LVEF (%) | -0.512 | 0.011 |
| Serum lactate | LVFS (%) | -0.493 | 0.014 |

Table Notes:

1.Sample size: 6 mice per group (SED-SHAM, AE-SHAM, SED-MI, AE-MI), totaling 24 mice with complete data.

2.Correlation strength definition: |r| ≥ 0.8 = very strong; 0.5 ≤ |r| < 0.8 = moderate; 0.3 ≤ |r| < 0.5 = weak.

3.Significance levels: P < 0.001 indicates a statistically highly significant correlation. P < 0.05 indicates a statistically significant correlation.

**Table S3. Linear regression analysis of lactate levels on cardiac function parameters**

| **Independent Variable** | **Dependent Variable** | **Regression Equation** | **R²** | **P-Value** | **95% Confidence Interval for β** |
| --- | --- | --- | --- | --- | --- |
| Heart lactate | LVEF (%) | LVEF=71.63−9.69× Heart lactate | 0.784 | <0.001 | [-11.93, −7.44] |
| Heart lactate | LVFS (%) | LVFS=38.78−5.76× Heart lactate | 0.774 | <0.001 | [-7.14, −4.39] |
| Serum lactate | LVEF (%) | LVEF=72.11−25.29× Serum lactate | 0.262 | 0.011 | [-44.06, −6.53] |
| Serum lactate | LVFS (%) | LVFS=38.67−14.59× Serum lactate | 0.243 | 0.014 | [-25.97, −3.21] |

Table Notes:

1. R² (Coefficient of Determination): Reflects the proportion of variance in the dependent variable explained by the independent variable.

2. Significance levels: P < 0.001 indicates a statistically highly significant correlation. P < 0.05 indicates a statistically significant correlation.
